# The comparison of white-to-white via triple person-times caliper measuring and machine-measuring in V4c implantable collamer lens implantation

**DOI:** 10.1038/s41598-024-64647-8

**Published:** 2024-06-16

**Authors:** Ting-Ting Dan, Tai-Xiang Liu, Zong-Ze Li, Ceng-Peng Liang, Fa-Yuan Li

**Affiliations:** 1 Department of Ophthalmology, Affiliated Hospital of Zunyi Medical University, Zunyi, 563000 Guizhou Province China; 2 Guizhou Eye Hospital, Zunyi, 563000 Guizhou Province China; 3 Guizhou Provincial Branch of National Eye Disease Clinical Research Center, Zunyi, 563000 Guizhou Province China; 4 Special Key Laboratory of Ocular Diseases of Guizhou Province, Zunyi Medical University, Zunyi, 563000 Guizhou Province China; 5 Department of Ophthalmology, The Affiliated Hospital of Zunyi Medical University, No. 149 Dalian Road, Huichuan District, Zunyi, 563003 Guizhou Province China

**Keywords:** ICL, White-to-white, Caliper, IOL-Master, Pentacam HR, UBM, Eye diseases, Medical research

## Abstract

This study aimed to compare the differences and characteristics of white-to-white (WTW) values obtained before V4c implantation using triple person-times caliper, IOL-Master 700, Pentacam HR, and UBM, and to assess their correlation with vaulting. A total of 930 myopia patients (1842 eyes) who were interested in undergoing ICL surgery were assessed before the procedure using various instruments. The WTW measurements were obtained using a triple person-times caliper, Pentacam HR, and IOL-Master 700, whereas the angle-to-angle (ATA) measurements were obtained using UBM. The size of the ICL was subsequently calculated using triple person-times caliper measurements. The vault of the ICL was assessed using Pentacam HR three months after the surgery. The WTW was determined to be 11.30 ± 0.29 mm, 11.43 ± 0.29 mm, and11.86 ± 0.38 mm, respectively, using the triple person-times caliper, Pentacam HR, and IOL-Master 700. The measurement of ATA was 11.57 ± 0.51 mm, as done by UBM. The ICL vault was measured to be 400.97 ± 198.46 µm when examined with Pentacam HR three monthsafter the procedure. The linear regression analyses of ICL size and WTW of triple person-times caliper, ICL vault and WTW were (R = 0.703, p < 0.001; R = 0.0969, p < 0.001) respectively. The highest correlation was found between IOL-Master and Pentacam HR (r = 0.766, p = 0.000). The lowest correlation was found between UBM and Pentacam HR (r = 0.358, p = 0.002). Bland–Altman analysis showed that the 95% limits of agreement (LoA) were the triple person-times caliper and Pentacam HR (– 0.573, 0.298) and the triple person-times caliper and UBM (– 1.15, – 0.605). This indicated a strong agreement between the triple person-times caliper and Pentacam HR and a lack of agreement between the triple person-times caliper and UBM. Triple person-times caliper measurements offer excellent maneuverability, practicality, and reliable outcomes for determining ICL vaults. Measurements obtained using the triple-person caliper were less differece than those obtained using the Pentacam HR.

## Introduction

Implantable collamer lens implantation (ICL) is a safe and effective intraocular refractive surgery that does not disrupt the structure of the cornea^1^. It closely mimics the normal physiology of the human eye, allowing the removal of glasses in individuals with high myopia. The parameters of the anterior segment of the eye must be measured to estimate the size of the ICL lens. Implantation of an appropriately sized lens is essential to avoid undesirable conditions, including displacement, high intraocular pressure, rotation, and improper vault fit^2,3^. In preoperative crystallographic calculations for ICL, the measurement of horizontal corneal diameter calipers is precisely described, and the establishment of uniform standards primarily depends on the examination experience. The estimation of V4c size traditionally depends on measuring the horizontal white-to-white (WTW) distance, to which an angle-to-angle (ATA) constant value is added, usually ranging from 0.5 to 1.0 mm^4^. Several articles^5–13^ have evaluated WTW using various automatic devices. Discrepancies have been reported between studies measuring horizontal WTW, mainly due to manual or automated measurement, with automated measurements yielding greater WTW^7^. Nevertheless, traditional caliper measurements are gradually being supplanted by machine measurements as inspection tools advance. It is accurate to state that the current formula for calculating crystal models is outdated. Additionally, caliper measurements lack comprehensive method descriptions, which may potentially contribute to their inability to be standardized. Maximizing the benefits of caliper measurements to ensure surgical safety is of interest to healthcare institutions with limited equipment availability or those in the initial implementation of the procedure due to policy and financial constraints. To achieve this objective, we conducted a statistical analysis of the triple person-times caliper measurements of WTW undertaken at our institution since 2017. We compared the results with those of various non-artificial methods and assessed the validity and usefulness of this enhanced caliper measurement method in relation to the postoperative outcomes of the patients.

## Methods

### Patients

A total of 1842 eyes from 930 myopic patients, with an average age of 24.58 ± 5.05 years (Range 18–39 years), were included in this study. All patients Underwent ICL-V4c implantation at the Department of Myopia Centre, Affiliated Hospital of Zunyi Medical College, from December 2017 to January 2023. Informed consent was obtained from all the patients before the study commenced. The retrospective study protocol was in accordance with the principles of the Declaration of Helsinki and was approved by the Ethics Committee of Zunyi Medical College. The inclusion criteria included myopic patients with stable refractive errors ranging from 0.5 to 18.00 D, corneal diameters greater than 10.8 mm, anterior chamber depths (ACD) greater than 2.8 mm, corneal endothelial cell counts higher than 2000 Cells/mm^2^ and no history of keratoconus or bacterial infection. The exclusion criteria included the following factors: a previous history of refractive instability, a history of ophthalmic surgery or surgical complications, any corneal or macular pathology, and diseases that cause vision loss, such as strabismus.

Additionally, cases related to systemic diseases, including immune disorders, connective tissue diseases, and depression, were excluded.

### WTW method

#### The triple person-times caliper

**Figure 1:**
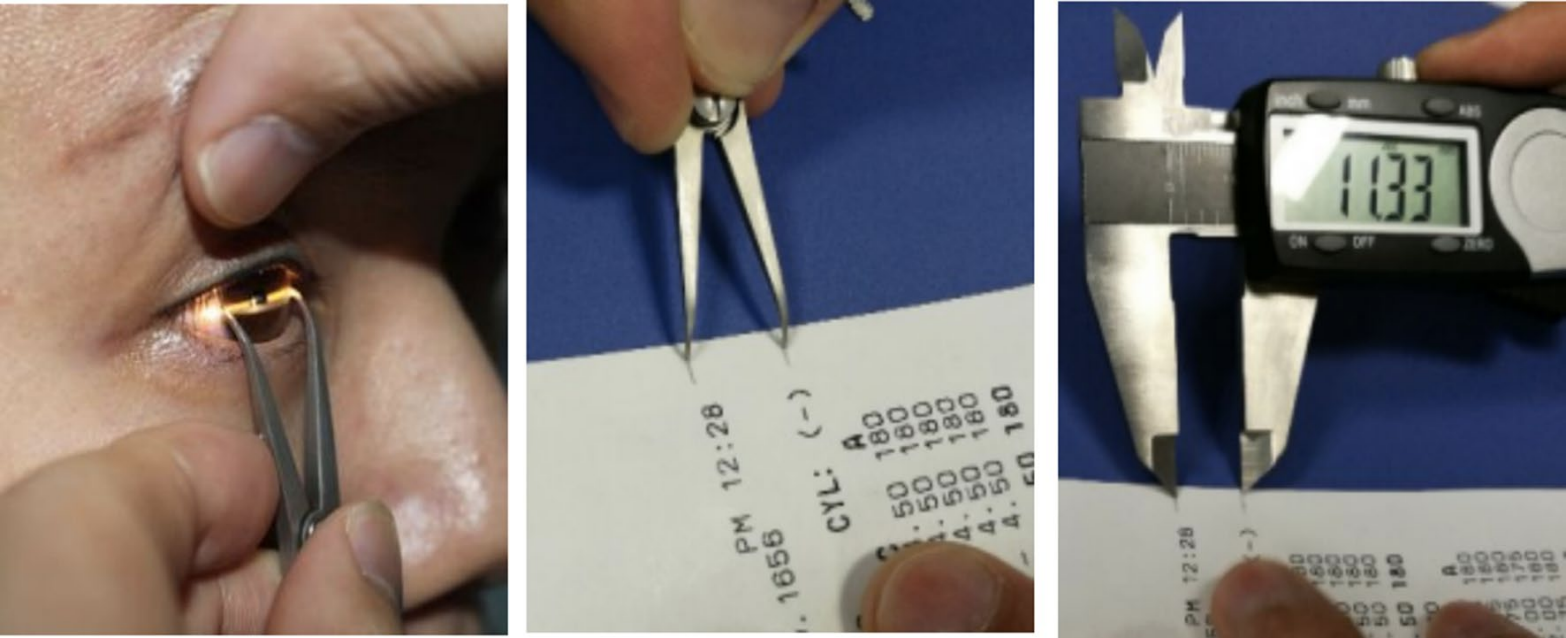
Method of triple person-time caliper measurements. Step 1: Measurement of the corneal diameter is taken from 3 to 9 points using an eye gauge caliper while the patient is seated at the slit lamp. Step 2: Tip of the eye gauge scratches on thermal paper. Step3: Digital vernier calipers measure the size of scratches.

A squint distance-measuring ocular caliper, digital vernier caliper, and thermal paper must be assembled first. The importance of triple-blind measurement was communicated to gain the patient’s cooperation and trust. Secondly, the calipers were calibrated after achieving local anesthesia with proparacaine hydrochloride (Alcon) (Fig. 1). After administering ocular anesthesia three times, the patient was comfortably placed in a seated position. Under bright light, the patient’s jaw was positioned on the mandibular rest, the forehead was placed against the frontal rest, and the patient was instructed to focus their attention on the cursor in front of them. Measurements were conducted monocularly with the dominant eye, and corneal diameter was assessed using ocular calipers. The maximum transverse diameter of the cornea was measured using the length of the three-point line connecting the corneal-scleral rims at 3 and 9 o’clock positions and the center of the pupil. This measurement was conducted after adjusting the slit lamp band to the horizontal position. The tip of the ocular caliper was used to create a mark on thermal paper, and the measurement of the mark was recorded using a digital vernier caliper. The next doctor recorded the measurements independently after zeroing the ocular caliper. The final result was determined from the average of the measurements taken by the three doctors.

The WTW of the Pentacam HR, the center value in dark light, was measured using the Scheimpflug analysis system, Pentacam HR (Oculus®, Wetzlar, Germany). It utilized a blue light diode and a rotating Scheimpflug camera for rapid, non-contact imaging of the anterior segment of the eye. The patient was instructed to look straight ahead at a blue circular fixation target using the Pentacam HR Rotating Scheimpflug Camera. The rotating camera acquired 25 images of the anterior segment in less than 2 s, while the imaging system automatically obtained the horizontal WTW measurement.

The IOL-Master 700 biometer (Carl Zeiss Meditec, Jena, Germany) is based on SS-OCT and relies on a wavelength of 1055 nm, a scanning depth of 44 mm, and a scan width of 6 mm. The resolution observed in the tissue was low. The size of the object is 22 µm, or it can be measured at a speed of 2000 A-scans per second. WTW is automatically computed after the device performs one and six measurements.

Ultrasound biomicroscope (Model SW-3200L, Tianjin Suowei Electronic Technology Co., Ltd.) is a high-resolution ultrasound technology application (30–50 Hz) that enables precise visualization of the atrial angle and truncal sulcus. ATA refers to the measurement of the diameter of the truncal sulcus in the supine position from 3 to 9 o’clock, with the pupil in its natural position.

#### Surgical techniques

All surgeries were performed by the same experienced surgeon (Dr. LTX). All measures were taken during the preoperative assessment. Finally, the definite size of the ICL lens to be implanted was determined in every case using the online calculator provided by the laboratory, which relies on the WTW values obtained from the triple person-times caliper measurements and ACD values obtained with IOL-Master 700 and Pentacam HR.

Each ICL surgery was performed through a 3.0 mm corneal incision at 11:00. After the administration of topical anesthesia using proparacanie (proparacaine hydrochloride, Alcon), the foldable V4 ICL was inserted into the posterior chamber through a 2.8 mm corneal incision with a particular design injector. The ICL was placed in the ciliary sulcus and rolled to a suitable angle using a triple person-times caliper. The remaining viscoelastic agent in the anterior chamber was subsequently completely removed by I/A with a balanced saline solution. Steroidal (0.1% fluorometholone, Santen, Osaka, Japan), antibiotic (0.3% levofloxacin, Santen, Osaka, Japan),NSAIDs (pranoprofen, Senju pharmaceutical, Japan), and sodium hyaluronate (1.7% sodium hyaluronate; Bausch & Lomb, China) were administered, and the doses progressively decreased over 1 month.

### Ophthalmologic measurements

Ophthalmologic measurements were evaluated preoperatively and three months postoperatively. The following data were collected: uncorrected distance visual acuity (UDVA), manifest refraction, corrected visual acuity (CDVA), endothelial cell count (ECC) using the Topcon Sp-2000p instrument (Topcon, Tokyo, Japan), intraocular pressure (IOP) using the TX-10 non-contact tonometer (Canon, Japan), axial length and anterior chamber depth (ACD) using the IOLMaster-500 instrument (Carl Zeiss Meditec AG, Germany). The center values in the dark light Pentacam HR and ACD in normal light were also measured using the Scheimpflug analysis system.

### Statistical analysis

Data are presented as mean ± SD. Sample size was calculated using G.Power software v 3.1 according to the standard deviation observed in a previous pilot study: the software indicated a minimum of 134 observations required for α = 0.05, risk β = 0.15 in bilateral contrast. Calculate only the right eye of each two myopia patients. A total of 930 right eyes from 930 patients were included in the statistics. All data were analyzed using SPSS Statistics 26.0 (SPSS Inc., Chicago, US). The Kolmogorov–Smirnov test was used to confirm data normality; independent t-tests were used for continuous variables with normality, and Wilcoxon tests were used to compare continuous variables without normality. The linear correlation coefficient was used to establish the correlation between the measurements obtained from each pair of devices. The Bland–Altman concordance method was used to determine the limits of agreement (LoA) at a 95% confidence level for pairings of measurements obtained from four different methods of WTW measurement.

### Ethics approval and consent to participate

The protocol adhered to the tenets of the Declaration of Helsinki, and Zunyi Medical University’s institutional ethics committee approved the study (Approval (20212)-524). A written informed consent regarding surgery was obtained from each patient before surgery. All methods were carried out in accordance with relevant guidelines and regulations.

## Results

Participants included 367 males and 563 females (Table1). preoperative spherical lens equivalent visual acuity was – 8.27 ± 3.26 D. The axial length of the eye was 26.59 ± 1.11 mm, and the depth of the anterior chamber was 3.18 ± 0.27 mm. The ratio of astigmatism to astigmatic lens was 810:1032 (78.48%). The sizes and numbers of ICLs implanted into the eyes were as follows: 12.1 (480), 12.6 (1104), 13.2 (248), and 13.7 (10). The preoperative uncorrected distance visual acuity (UDVA) was 1.39 ± 0.27, and the CDVA was 0.069 ± 0.13. The postoperative CDVA was 0.07 ± 0.13, and the UDVA was – 0.02 ± 0.11. There was a significant difference before and after the procedure in UDVA (t = 63.93, p = 0.00) and CDVA (t = 13.32, p = 0.00). ICL implantation effectively improved UDVA and was superior to corrected CDVA.

**Table 1:** Demographics and Clinical characteristics.

Parameters	Mean ± SD	Range
Sex (M:F)	367/563	
T/I (%)	810/1032 (78.48%)	
Age (a)	24.4728 ± 5.27695	18, 39
ICL size (mm)	12.1 (480), 12.6 (1104), 13.2 (248), 13.7 (10)	
SE (D)	– 8.2685 ± 3.263	− 3, − 18
IOP (mmHg)	13.6759 ± 6.08334	9.7, 23.0
AL (mm)	26.5912 ± 1.113	24.66, 29.87
ACD (mm)	3.183 ± 0.269	2.78, 4.09
Corneal thickness (μm)	513.743 ± 31.659	440,596
PD (mm)	3.127 ± 0.564	1.99, 4.96
Pre-UDVA	1.3985 ± 0.274	0.82, 270
Pre-CDVA	0.0689 ± 0.1343	0.00, 0.92
Post-CDVA	0.016 ± 0.107	− 0.18, 0.78
Post-UDVA	− 0.0159 ± 0.108	− 0.18, 0.6

Sex F:M, the number of female and male of patients; T/I, the number of Toric Implantable Contact Lens and Implantable Contact Lens; Age, the years of patients; SE, spherical equivalent; IOP, intraocular pressure; AL, the ocular axis length; ACD, anterior chamber depth; PD, pupil diameter; UDVA, uncorrected distance visual acuity; CDVA, corrected visual acuity; Pre, preoperative.

**Figure 2:**
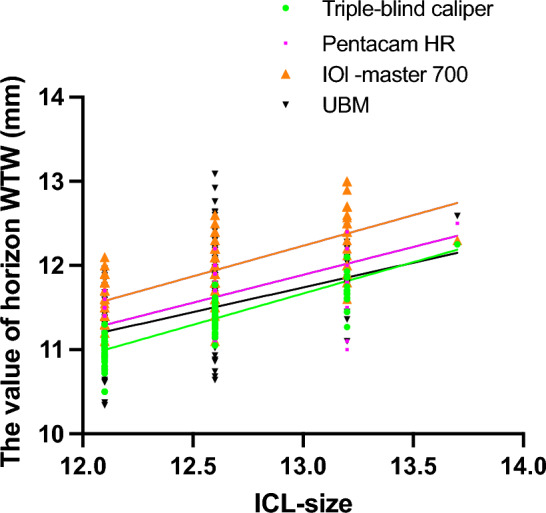
The linear regression lines of ICL size and horizon WTW of four methods.

**Figure 3:**
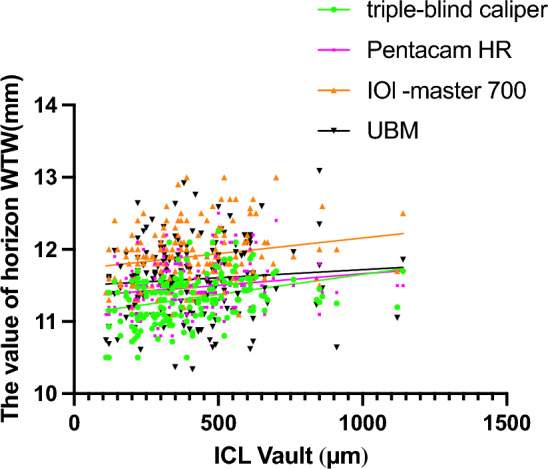
The linear regression lines of ICL vault and horizon WTW of four methods.

The mean horizontal corneal diameter was found to be 11.30 ± 0.29, 11.43 ± 0.29, and 11.86 ± 0.38 mm using the triple person-times caliper, Pentacam HR, and IOL-Master 700, respectively (Table 2). The ATA was determined to be 11.57 ± 0.51 mm, as measured by UBM. The caliper measurement indicated that the size of the ICL was 12.0 ± 0.5 mm. The arch height measured by Pentacam HR after a three-month intervention was 400.97 ± 198.46 μm (Range 110.00–1140.00 μm). The linear regression analyses of the ICL size and the WTW measurements from four methods were as follows: triple person-times caliper (R = 0.703, p < 0.001), Pentacam HR (R = 0.435, p < 0.001), IOL-Master 700 (R = 0.4772, p < 0.001), and UBM (R = 0.2209, p < 0.001) respectively (see Fig. 2). The linear regression analyses of the ICL vault and the WTW of four methods, including triple person-times caliper (R = 0.0969, p < 0.001), Pentacam HR (R = 0.0305, p = 0.0208), IOL-Master 700 (R = 0.0411, p = 0.0075), and UBM (R = 0.0668, p = 0.3057) respectively (Fig. 3). All of them indicated that the triple-blind method had a higher linear correlation than the other methods regarding ICL size and ICL vault.

**Table 2:** The WTW value of four methods and vault.

White-to-white	Mean ± SD	Range
Triple person-times caliper (mm)	11.30 ± 0.29	10.50, 12.25
Pentacam HR (mm)	11.43 ± 0.29	10.80, 12.50
IOL -Master 700 (mm)	11.86 ± 0.38	11.10, 13.00
UBM (mm)	11.57 ± 0.51	10.34, 13.09
Spherical -ICL (D)	− 8.02 ± 2.69	− 18.00, 3.60
Cylindrica-ICL (D)	− 1.60 ± 1.23	− 5.00, 4.00
Spherical (D)	− 8.15 ± 3.00	− 18, − 3.0
Cylindrica (DC)	− 1.09 ± 1.18	− 5, − 1.5
pre-PD (mm)	3.13 ± 0.59	1.99, 4.96
Post-PD (mm)	3.27 ± 0.63	2.07, 5.65
pre-ACD (mm)	3.19 ± 0.25	2.78, 4.09
post-ACD (mm)	3.18 ± 0.38	2.16, 4.41
post-SE (D)	0.112 ± 0.69	− 3.5, 1.2
Valut (μm)	400.97 ± 198.46	110.00, 1140.00

Pre, preoperative; post, postoperative; PD, pupil diameter; ACD, anterior chamber depth; SE, spherical equivalent.

Table 3 shows the mean value and statistical significance of the differences in horizontal diameter measurements for each pair of variables. There were statistically significant differences observed in the horizontal corneal diameter measurements between the following pairs: triple person-times caliper—Pentacam HR (p < 0.001), triple person-times caliper—IOL-Master 700 (p < 0.001), triple person-times caliper—UBM (p < 0.001), Pentacam HR—IOL-Master 700 (p < 0.001), and Pentacam HR—UBM (p = 0.01), and IOL-Master 700—UBM (p < 0.001). The strongest correlation was observe between IOL-Master and Pentacam HR (r = 0.828, p = 0.00), and the lowest correlation was observed between UBM and Pentacam HR (r = 0.364, p = 0.001).

**Table 3:** The paired samples test and pearson correlation of WTW value by four methods.

	Paired samples test				Pearson correlation	
	Mean	SD	T	p	r	p
Triple person-times caliper—Pentacam HR	− 0.145	0.197	− 6.524	0.000	0.766	0.000
Triple person-times caliper—IOL-Master 700	− 0.558	0.254	− 19.649	0.00	0.738	0.00
Triple person-times caliper—UBM	− 0.265	0.4629	− 4.996	0.00	0.44	0.00
Pentacam HR—IOL-Master 700	− 0.407	0.1996	− 18.107	0.00	0.828	0.00
Pentacam HR—UBM	− 0.131	0.489	− 2.319	0.01	0.358	0.002
IOL -Master 700—UBM	0.291	0.511	4.974	0.00	0.364	0.001

**Figure 4:**
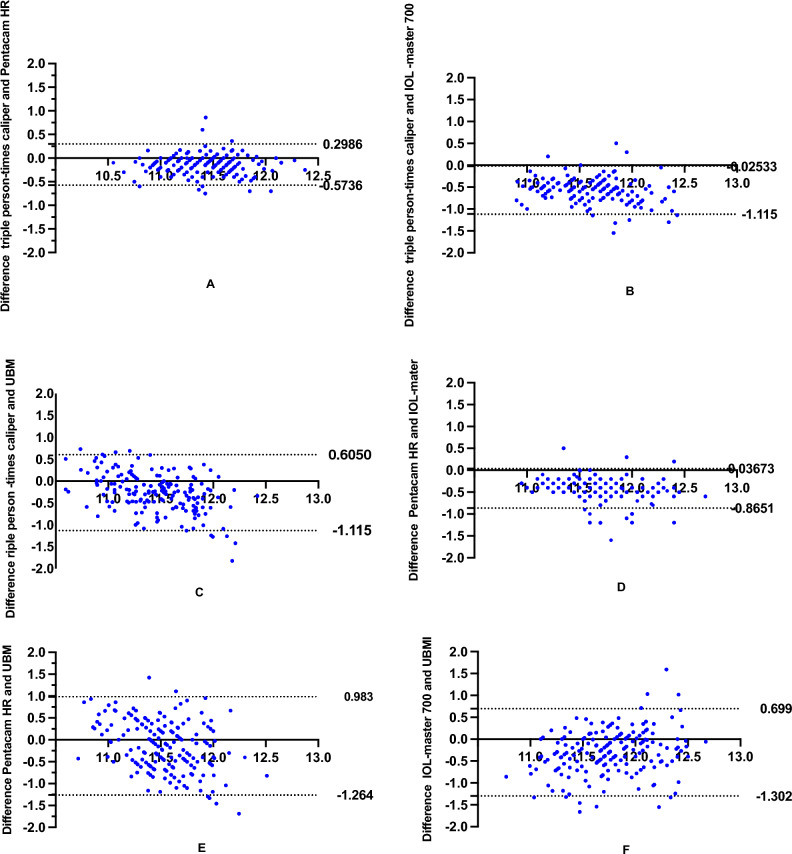
The Bland–Altman graphical representation between the means of each pair of measures versus the difference between devices. (**A**) Difference vs. average: Bland–Altman of triple-blind caliper and Pentacam HR. (**B**) Difference vs. average: Bland–Altman of triple person-times caliper and IOL—master 700. (**C**) Difference vs. average: Bland–Altman of triple person -times caliper and UBM. (**D**) Difference vs. average: Bland–Altman of Pentacam HR and IOL-mater. (**E**) Difference vs. average: Bland–Altman of Pentacam HR and UBM. (**F**) Difference vs. average: Bland–Altman of IOL-master 700 and UBM.

The Bland–Altman analysis identified that the 95% LoA between the triple person-times caliper and the Pentacam HR was − 0.57 mm and 0.29 mm, respectively (Fig. 4A). The maximum absolute difference of 0.13 mm indicated a strong agreement between the two methods. This value falls comfortably within the clinically acceptable maximum range of 0.5 mm. Similarly, the 95% LoA values between the triple person-times caliper and the IOL Master were − 1.12 and − 0.03 mm (Fig. 4B), with a maximum absolute difference of 0.57 mm. Which indicated a lack of agreement between the two methods. This is unacceptable, as the maximum clinically acceptable range is 0.5 mm. The 95% LoA of the triple person-times caliper and UBM were − 1.12 and 0.61 mm (Fig. 4C),with a maximum absolute difference of 0.26 mm. The 95% LoA of Pentacam and IOL-Master were − 0.87 and 0.04 mm (Fig. 4D), with a maximum absolute difference of − 0.41 mm, suggesting a strong agreement between the two measurement methods. The difference in measurement between IOL-master and Pentacam HR measurements was 0.037, which was greater than the reported in the literature^16^. The 95% LoA values for Pentacam and UBM were − 1.26 and 0.98 mm (Fig. 4E), respectively, with a maximum absolute difference of − 0.14 mm, indicating a strong agreement between the two methods. The 95% LoA values for IOL-Master and UBM were − 1.30 and 0.69 mm (Fig. 4F), respectively, with a maximum absolute difference of − 0.30 mm, demonstrating a strong agreement between the two methods.

## Discussion

ICL implantation has rapidly increased from 1 million in 2019 to 2 million in 2022 worldwide^14^, especially in developing countries, as the prevalence of high myopia has grown. It is essential to select the optimum ICL size to prevent the risk of short-term and long-term post-surgical complications^15–17^. Conventionally, only two parameters are considered in the manufacturer’s ICL size recommendation: WTW and ACD (Visian ICL product Information: Visian ICL For Myopia). According to our results, neither WTW measured by any instrument nor ACD was a reliable predictor of postoperative vaulting, which was the consensus of many similar studies^18–20^. Regarding WTW, significant discrepancies were observed in the values obtained from the different devices, indicating low correlation levels^21^. Ideal ICL surgery requires precise preoperative parameter measurements. The measurement of horizontal corneal diameter using calipers is not described in detail in the preoperative crystallographic calculations for ICL. The FDA uses orbscan for preoperative WTW measurements in the United States; however, such conditions are not readily available in many regions and hospitals, especially in developing countries. Therefore, it is important to identify a simple and economical method for corneal cataract measurement. According to the literature, traditional caliper measurements are unreproducible because of human factors^22,23^, but we did not encounter this issue.

This study focused on a retrospective analysis to investigate the differences in the methods used to measure the WTW distance. These methods included the triple person-times caliper, IOL-Master 700, Pentacam HR, and UBM with different scales. Additionally, the study aimed to evaluate the feasibility of using these methods for ICL-V4c implantation. This will provide a foundation for selecting more effective measurement methods.

The Pentacam HR uses optical slit rotation. The camera captures images of the anterior segment from various angles and processes them using computer technology to create its 3D stereogram. This allows measurements of the height of the front and back surfaces, corneal refraction, corneal thickness, corneal WTW, ACD, and more. It has the advantage of being certain such as set the thinnest point, and do not contact the cornea directly. The disadvantage of this method is that it is affected by corneal and eyelid transparency, which prevents occlusion and renders it ineffective on the tear film. The average WTW measurement obtained from the Pentacam HR was 11.30 ± 0.29 mm. The value estimated by Pentacam HR is lower than the value estimated by other measurement methods.

The IOL-Master 700 biometer is based on SS-OCT technology. The device operates at a specific wavelength of 1055 nm and has a scanning depth of 44 mm and a scanning width of 6 mm. The tissue resolution is 22 µm, and the measurement speed was 2000 A-scans/s. The WTW and ACD distances are calculated automatically by the device after completing one and six measurements, respectively. The average WTW measured by the instruments was 11.86 ± 0.38 mm, and the estimated value was smaller than Calvo Sanz’s 12.05 ± 0.30^24^. In accordance with other studies, the IOL-Master produced the largest numbers of measurements, whereas the triple person-times calipers yielded the smallest^25–30^. The WTW measurements obtained using the Pentacam HR Ocular Nodule Analyzer System and the calipers were relatively close, and the differences were not statistically significant (p > 0.05). However, the independent samples t-test results for the differences between the four methods were also not statistically significant. The difference in WTW between these two measurements and the other three was statistically significant (all p < 0.05). A paired t-test was conducted to analyze the differences between the four methods in paired comparisons. The strongest correlation was observe between IOL-Master and Pentacam HR, and the lowest correlation was observed between UBM and Pentacam HR. This difference can be attributed the varying to measurement methods used, as the IOL-Master and Pentacam HR rely onoptical measurements, while UBM utilizes ultrasound measurements. Furthermore, the truncated sulcus at the measurement point of the UBM does not precisely correspond with the horizontal angular diameter of the sclera. Additionally, it should be noted that the truncated sulcus at the point of measurement of the UBM is not exactly equal to the horizontal angular diameter of the sclera^31^. In the group with an ACD > 3.5 mm, there was no significant correlation between WTW and sulcus to sulcus (S–S)^32^. UBM measurements require using an eye cup to immerse the eye in water. This will exert pressure on the eyeball, causing it to deform and affecting the measurement results.

A Bland–Altman analysis indicated a strong agreement between the triple person-times caliper and Pentacam and a lack of agreement between the triple person-times caliper and IOL-Master. It also revealed a strong agreement between the Pentacam and IOL-Master, similar to that between IOL-Master and UBM.

Although some scholars use machine-measured WTW to extrapolate new formulas to predict arch heights^8,11^, traditional manual calipers offer advantages. It is commonly believed that manual measurements are susceptible to errors and personal influence. A meta-analysis showed that 83.5% and 86.5% of eyes could attain a clinical vault ranging from 250 to 1000 μm, respectively, when the ICL size was determined using the WTW sizing method and S–S sizing method, respectively^36^. To ensure the accuracy and reproducibility of the data, we established uniform standards and then conducted department-wide training and education. Inparticular, accurate identification of the position of the WTW under the slit lamp and microscope, along with the pupil area from 3 to 9 o’clock, can effectively prevent errors that may occur in WTW measurements after eye rotation.Measurements were repeated three times by the surgeon and two additional physicians. The calipers were reset to zero after each measurement to ensure the accuracy and independence of the three individuals. Electronic vernier calipers measure distances more accurately than direct degrees. The measurement data can be precise to two decimal places, compared with direct measurement, which is accurate to one decimal place. Additionally, they can be used with thermal paper to avoid prolonged tension on the patient’s eyeball and enable repeated measurements without direct contact. However, calibration of the measurement is inconvenient. An average value should be obtained from the measurements of the same patient from multiple physicians, ensuring that no special circumstances are overlooked. Continuous feedback should be accumulated to ensure a consistent and direct measurement experience. Upon reaching the critical value, the surgeon must integrate the caliper measurements with the machine data to decide the lens type and its preparation.

The triple person-times caliper measurements are highly accurate and reproducible. First, ophthalmic calipers offer a more precise measurement of the shape of the eye, thereby enabling measurements to be taken while observing the eye’s condition. This prevents eyelash interference, corneal vascular opacities, and other factors^22^. Second, vernier calipers provide a significantly higher accuracy of 0.01 mm compared with the 1-mm accuracy of triple-person-times calipers. Additionally, ophthalmic calipers contribute to patient safety by preventing scratches on the eye caused by the caliper’s tip, thereby eliminating the need for repetitive procedures. Furthermore, the use of ophthalmic calipers facilitates the comparison of measurements, which assists in identifying any discrepancies and their origins. Utilizing the combination of the two caliper methods, examiner can facilitate to judge the reliability of the results by the slit lamp observation of ocular, and sitting measurement can effectively exclude the effect of prone eye rotation on the axial direction of the eye. Eye movement during the transition from the seated to the prone position ranges from 2 to 14 degrees inward or outward^33^. Furthermore, the accuracy of the results was improved by consistent training in the measurement criteria and the blinding of three examiners, indicating that they were unaware of each other’s calculations. Finally, the economic benefits associated with caliper measurements should not be disregarded.

The vault consistently exceeded 400.97 ± 198.46 within the range of 110–1140 µm. According to a previous study, the minimum vault required distance ranged from 90 to 230 µm^13,15^, and this value decreased to 52 µm in patients over 45 years of age^14^. A very high vault (> 1000 µm) can give rise to complications such as pupillary block and angle closure, causing glaucoma^16^. In this study, the lowest measurement was determined to be 110, and the highest was 1140, as STS varies with WTW of anatomically aberrant structures, all of which alter the ICL size.

A low overall incidence of 0.32% was observed after ICL-V4c implantation. This was significantly less than the 0.8% Chinese average and the 0.25% of the Shanghai Fudan University Eye, Ear, Nose, and Throat Hospital^34^. AlSabaani NA had 3.8% exchange rate^35^. Packer et al. summarized a 0.47% incidence of secondary surgical intervention in 2970 eyes from 28 articles^36^. In a multicenter study, Kamiya^37^ observed 351 eyes for 12 months and reported that two eyes were exchanged because of incorrect initial sizing or power. In this study, six eyes were exchanged due to the ICL size; one patient had a very high value of 1240 µm, and two patients had low arches of less than 120 µm, although they did not exhibit any complication. Three patients had astigmatic axial rotation despite the value falling within the normal range. The primary reason for this condition was the anatomical difference between the truncus and the lens. Our study had limitations like crystalline lens rise^38^, ciliary body cyst, atrial angle, and crystal haptic^39^, which should be considered in a few patients with suboptimal arch height.Despite the abundance of software and instruments available for calculating arch heights, we continued to rely on caliper measurements and achieved good arch heights postoperatively.

In conclusion, the triple person-times caliper, Pentacam HR, IOL-Master, and UBM are indispensable for measuring the WTW distance. The triple person-times caliper measurement of WTW has demonstrated accuracy, reproducibility, reliability, and irreplaceability in V4c ICL surgery. The triple-person caliper measurements provide excellent maneuverability, practicality, and dependability for determining ICL vaults. Measurements obtained using the triple-person caliper were less different from those obtained with the Pentacam HR.

## Data Availability

The datasets generated during and/or analyzed during the current study are available from the corresponding author on reasonable request.

## Abbreviations

ICL: Implantable Contact Lens
TICL: Toric Implantable Contact Lens
AL: Axis length
UDVA: Uncorrected distance visual acuity
CDVA: Corrected distance visual acuity
WTW: White-to-white
ATA: Angle-to-angle
STS: Sulcus to Sulcus
ECC: Endothelial cell count
IOP: Intraocular pressure
ACD: Anterior chamber depth
PD: Pupil diameter
